# Analyzing medical device connectivity and its effect on cyber security in german hospitals

**DOI:** 10.1186/s12911-020-01259-y

**Published:** 2020-09-29

**Authors:** Markus Willing, Christian Dresen, Uwe Haverkamp, Sebastian Schinzel

**Affiliations:** 1 University of Münster, ■■■, Germany; 2 Münster University of Applied Sciences, ■■■, Germany

**Keywords:** Networked medical devices, IT-security, Critical infrastructure, Germany

## Abstract

**Background:**

Modern healthcare devices can be connected to computer networks and many western healthcare institutions run those devices in networks. At the same time, cyber attacks are on the rise and there is evidence that cybercriminals do not spare critical infrastructure such as major hospitals, even if they endanger patients. Intuitively, the more and closer connected healthcare devices are to public networks, the higher the risk of getting attacked.

**Methods:**

To asses the current connectivity status of healthcare devices, we surveyed the field of German hospitals and especially University Medical Center UMCs.

**Results:**

The results show a strong correlation between the networking degree and the number of medical devices. The average number of medical devices is 25.150, with a median of networked medical devices of 3.600. Actual key users of networked medical devices are the departments Radiology, Intensive Care, Radio-Oncology RO, Nuclear Medicine NUC, and Anaesthesiology in the group of UMCs. In the next five years, the usage of networked medical devices will increase significantly in the departments of Surgery, Intensive Care, and Radiology. We detected a strong correlation between the degree of connectivity and the likelihood of being attacked.The survey answers regarding the cyber security status reveal a lack of security basics in some of the inquired hospitals. We did discover successful attacks in hospitals with separated or subsidiary departments. A fusion of competencies on an organizational level facilitates the right behavior here. Most hospitals rated themselves predominantly positively in the self-assessment but also stated the usefulness of IT security insurance.

**Conclusions:**

Concluding our results, hospitals are already facing the consequences of omitted measures within their growing pool of medical devices. Continuously relying on historically grown structures without adaption and trusting manufactures to solve vectors is a critical behavior that could seriously endanger patients.

## Background

Healthcare institutions worldwide are facing a growing threat of cyber attacks. Many networked medical devices were found to contain critical security vulnerabilities [1]. Furthermore, medical devices tend to have a long life-span. Medical institutions may operate medical devices well beyond the point in time, at which the producer will not issue security patches. Due to these vulnerable systems in hospital networks, healthcare is among the most attacked sectors globally [2], and there are several public known attacks on healthcare infrastructure [3, 4].

A way to prevent these devices from attacks is to asses and shield them from attackers, e.g., by operating them in a restricted environment. In this paper, we study how German hospitals manage networked medical devices and how their respective approach addresses common cybersecurity threats. Concretely, we answer the following questions:

- What is the degree of connectivity of medical devices in German university medical centers and how is network connectivity and medical digitization developing in different departments?
- How is a growing network degree within active medical devices affecting cyber security in German hospitals?
- How do organizational structure and self-assessment reflect the particular threat level?

In Germany, there are overall 1943 hospitals [5]. The hospitals having a minimum of 30.000 inpatient cases per year are a member of the Critical Infrastructure Protection CIP according to the German IT Security Act of 2015 and the Critical Infrastructure Regulations for Hospitals of CIP published in 2017 [6]. The exact number of CIP hospitals is kept confidential, but we estimate it to be a total of 80-110 Hospitals [7] in Germany. From those hospitals, 36 are University Medical Center UMCs.

In 2017, the total patient case number in all German hospitals was 19,4 million fully inpatient cases [8]. The 36 UMCs alone covered about 10.8 million outpatients and about 1.9 million inpatients. This results in 9.7% of the German inpatient healthcare treatment covered by 1.85% of the hospitals [9].

To get an overview of the security status on networked medical devices in German Hospitals, it is essential having insights into the network situation of specific hospitals. Unfortunately, to the best of our knowledge, there is no public dataset about the degree of medical device connectivity in hospitals available.

### 1.1 Cyber security

Medical devices offer critical services and process sensitive data of patients with high protection requirements. In particular, the security of data and services is split into three distinct properties ([pp.8], [10]):

- Confidentiality: The confidentiality of the data must be protected for stored and transmitted data from the access of authorized persons.
- Integrity: The integrity of data in transit and at rest must be protected from unintended or unauthorized modification. In the case of integrity loss, this modification should be detected immediately.
- Availability: The availability of the data must be protected for stored and transmitted data to ensure access for all authorised persons, when it is needed.

The term cyber security stands for the strict adherence of these three properties within the cyber space, which spans over all digital devices that are connected to the Internet or to private networks with direct or indirect Internet connections. Note that we define a private network to be indirectly connected to the Internet if devices like laptops or USB drives are used in the private network as well as the Internet. An example is a USB drive that is used to move software updates from an Internet-connected computer to a computer within the confined private network.

An incident in which a remote attacker negatively affects at least one of these three key properties is called an cyber security incident.

### 1.2 Cyber security of networked devices

To attack non-networked devices, an attacker must be on-site and perform the attack manually, i.e. change device configurations by pushing buttons on the device’s user interface, or to copy, to steal or to modify patient’s data.

This changes once a device becomes networked. A networked device can be attacked remotely if the attacker manages to connect to it. In many cases, network segmentation and perimeter security protect external attackers from connecting to medical devices directly. However, a single exposed system is enough for an attacker to get a foothold within the local network. The attack can move laterally between different networks if the compromised machine is part of both networks. The more systems are connected to linked networks, the more potential targets are available to an attacker. ([p.637], [11]).

### 1.3 Related work

Due to the increasing trend of network usage in German hospitals, medical devices are becoming more and more endangered to threats of cyber criminality [12, 13]. One of the most severe cases in the past years was the non-targeted WannaCry-infection in April 2017. The affected medical devices caused several hospitals to discontinue their service, mainly in the UK[14]. Due to a hospital study in 2017, 64% of all German hospitals were victims of cybercrime [15].

Responding to this trend, the cyber security research community is starting to focus more on the networked healthcare sector, revealing a variety of vulnerabilities inside medical devices [16, 17].

A significant part of the networked system in a modern hospital consists of desktop workstations and networked medical devices. The responsibility for correct operation is historically separated between a central department of Information Technology IT and a central department of Medical Technology MT within the organization structure. Accordingly, the desktop workstations are managed by a central IT department, while medical devices are maintained by the producer and the central department of MT. As medical devices have to comply with regulations, including certification renewal for every change, patching of those devices generates a much higher effort than common proposed devices like desktop workstations[18, p.41].

UMCs are profitable targets for ransomware attackers due to their large sales volume of 22.1 billion Euros per year (2015) [19]. This leads to an average volume of about 614 million Euros per hospital. Larger hospitals even exceed one billion Euros per year. The Charite in Berlin, for example, states a yearly volume of 1.8 billion Euro [20].

A study in the Critical Infrastructure Protection CIP sector was conducted by the Bundeswehr University Munich and the Friedrich-Alexander University Erlangen-Nuremberg in 2018 [21]. This study surveyed the entire CIP sector, including a minor part of healthcare institutions, but not focused on healthcare in particular. Nevertheless, this study could serve as a basis for comparison.

Due to the 2019 hospital report of the WidO, German hospitals are behind their international counterparts. They are blaming a missing culture of innovation and a backlog of investments for this behavior[22]. One important factor is the lack of available stable broad band connections in Germany. Furthermore, the standardizing process for digital patient data and interoperability of medical devices is not in a sufficient state to build digital solutions without limitations. This can lead to a low adoption rate within pilot projects and a general user and management scepticism concerning the digital change in German hospitals [23].

## Methods

To measure the medical device connectivity and level of preparation against cybersecurity incidents in German hospitals, we developed 11 questions as a foundation for structured interviews (see Additional Files). The interview is addressed to Information Technology IT risk managers and leading IT and Medical Technology MT representatives of German hospitals. We conducted the interviews via telephone. The Questionnaire is attached in the Additional file 1.

The questions are separated into three categories:

- Demographic information about the hospital and its medical devices,
- The hospital’s degree of connectivity of medical devices, and
- Information about the cybersecurity status.

In the first Category on the hospital’s demographics (1) the questions aim at acquiring the total number of medical devices inside the hospital and whether the hospital is a member of Critical Infrastructure Protection CIP.

The definition of medical devices considered in this study is based on the German act of medical devices [24, §3(1)]. It characterizes a product as a medical device, when it is primarily used for the detection, prevention, monitoring, treatment, alleviation or compensation of disease, injuries or disabilities, for the examination, replacement or modification of the anatomical structure or a physiological process, or for conception regulation.

In our study we looked particularly at active medical devices. These are devices that are depending on an external energy source, including but not limited to: Radiological equipment, laboratory diagnostic systems, monitoring and life support systems, and all kinds of electronic treatment and supporting systems.

Furthermore, we collect the positions of the department of MT and the department of IT within the hospital’s organizational structure. Historically, those departments are separated from each other [25]. The level of connectivity of networked medical devices rises, and as many of them are connected to IT networks, the responsibilities of both departments increasingly overlap. This creates incentives in modern hospitals to merge both departments, and we ask for detailed information about these two departments in hospitals. The second Category (2) asks questions about the degree of connectivity of medical devices. This is done using four questions regarding the currently existing and the estimated future level of connectivity of medical devices per hospital department as well as for all medical devices combined in that hospital. The last Category (3) of the questionnaire aims at gathering information on general IT security aspects inside the hospital. It also assesses the hospital’s preparation for an information security incident and if the hospital leadership considers cyber insurance useful for hospitals. Finally, the questionnaire asks for information on past security incidents that affected the hospital‘s networked medical devices and their consequences.

Structured interviews can be prone to errors, especially if they are conducted by telephone. Possible sources of errors, for example, may result from a different understanding of questions or terms as well as different counting of devices. Furthermore, it is crucial for the correctness of the data to have the right contact person providing reliable information. To deal with those caveats, a basic understanding of the crucial terms was established during the interview process. Furthermore, we checked the plausibility of the given information using data provided by the German Federal Statistical Office.

## Results

Initially, we directly contacted 50 German hospitals via email and asked for their participation in the survey. We did focus on the hospitals with the largest capacity of inpatient care. Furthermore, the German hospital federation sent our survey questions to the boards of the German hospital associations, but we never received an answer. After 18 months, 20 (40%) hospitals responded. Out of these, in total, four hospitals declined the interview request. Two from this group did not mention a reason; one blamed capacity problems, and one hospital was not able to provide the required information about their infrastructure. We conducted the interviews with the 16 remaining hospitals in 12 weeks after they agreed to the interview.

As a result, we were able to create a data-set of 16 completed interviews with hospital representatives. From these 16 hospitals, there are 11 University Medical Centers UMCs, three Standard Care Hospitals SC, which are part of Critical Infrastructure Protection CIP, and two non-CIP SCs, as described in Table 1. In the following subsections, the results of the interview are described per category.

**Table 1:** This table shows our pool of responding hospitals. It consists of 11 UMC and 5 SC hospitals. The group of SC hospitals is divided into members of CIP and non-members

	**CIP**	**Non-CIP**
UMC	Standard Care	Standard Care
11	3	2

### 3.1 Category (1): demographic information on hospitals

**General information regarding the hospital and medical devices**

**Fig. 1:**
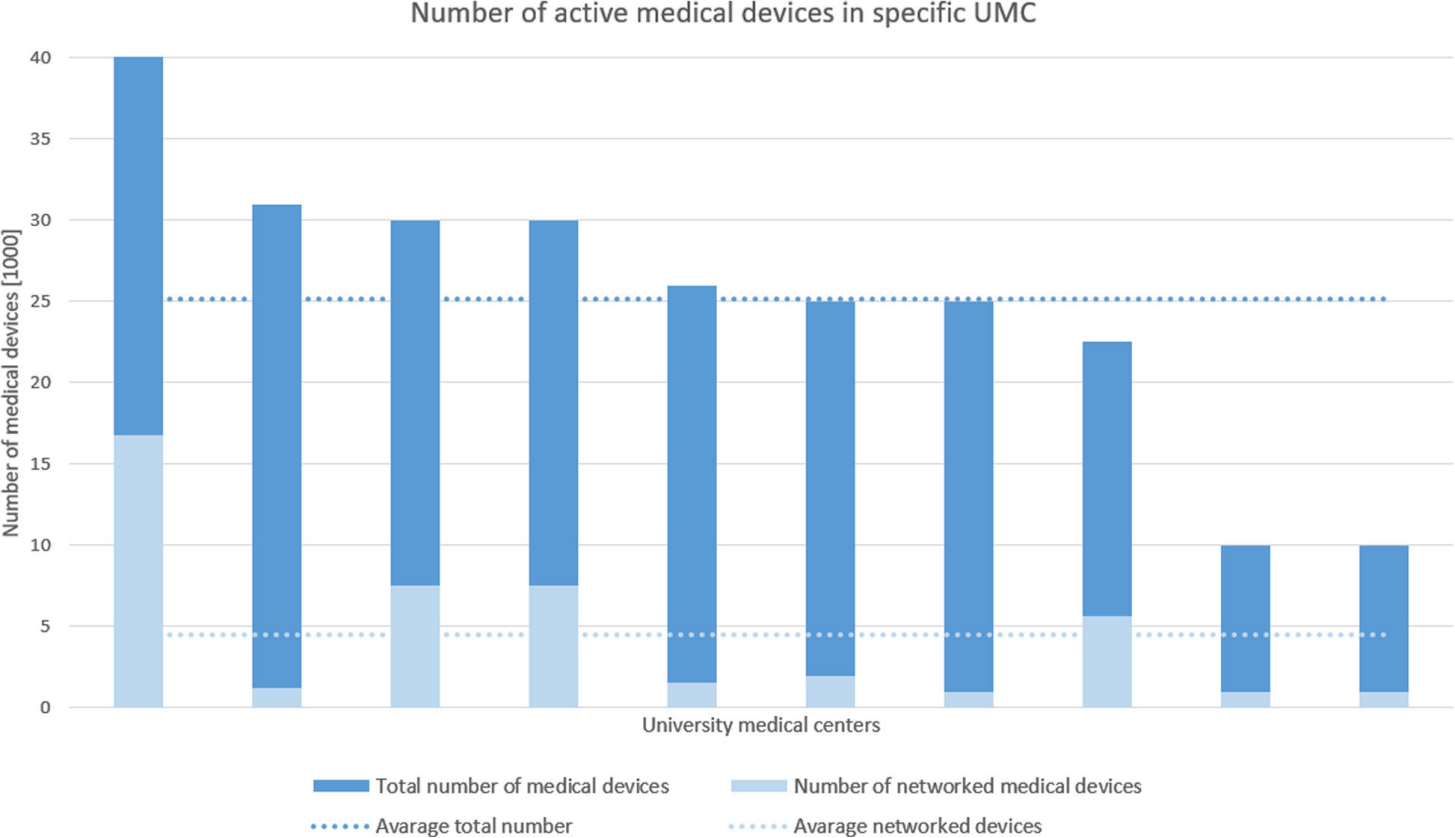
This figure illustrates the total number of active devices and their networked proportion. The amount ranges between 42.000 for large hospitals and about 10K medical devices. The average total number of medical devices amounts to about 25.000 and the mean of networked medical devices is about 3.600

**Fig. 2:**
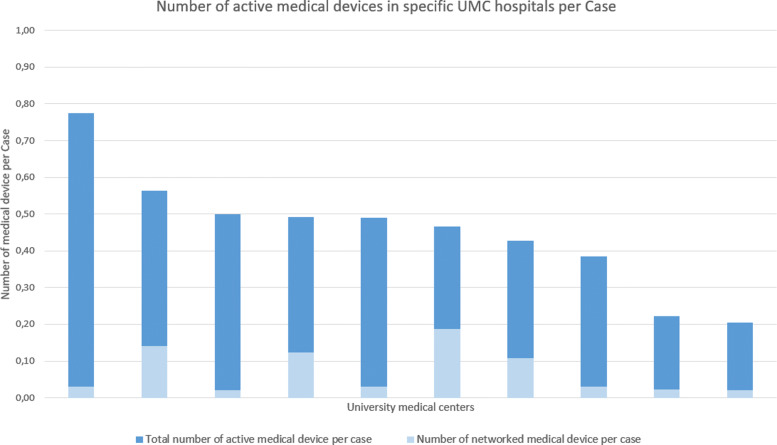
This figure presents the number of active medical devices standardised to the hospital specific number of inpatient cases per year. It shows a range between about 0.8 to 0.2 devices per case. Seven hospitals are in the range between 0.6 and 0.4 devices per case. The number of networked medical device per case ranges between 0.02 and 0.19

An essential indicator for estimating the information security status of hospitals is the number of medical devices, especially the ratio of networked ones. Figure 1 and Table 2 show the interview results for this question. The mean of medical devices in the group of UMCs amounts to 25.000 devices. Two hospitals stated a substantially lower value and one a higher amount of medical devices. The median totals to 25.500 devices. To get a more comparable result, we normalized the number of medical devices using the hospital’s number of cases per year documented in the latest quality reports of the relevant hospitals. The data is visualized in Figure 2. For anonymity reasons, the data in Figure 2 is not in the same order as in Table 1 but ordered from high to low instead. This results in a total of seven hospitals ranging between 0,4 and 0,6 devices per case. The remaining three hospitals deviate from this group with one much higher (0,78%) and two substantially lower (0,22% and 0,2%). The number of networked medical devices per case has a median of 0,03.

**Table 2:** This table presents the quantitative data of the inquired UMC hospitals regarding the active medical devices. The table also presents the ar. average, median and standard derivation within the networked and total number of medical devices. As described in “Discussion” section, the sixth hospital is treated as an outlier due to its implausible high networking degree

**Hospital**	**Total number of devices**	**Number networked medical devices**
1	31.000	1.240
2	22.500	5.630
3	25.000	1.000
4	30.000	7.500
5	26.000	1.560
6	42.000	(16.800)
7	30.000	7.500
8	25.000	2.000
9	10.000	1.000
10	10.000	1.000
Ar. Average	25.150	4.500
Median	25.500	3.600
Standard derivation	9.610	2.860 (exkl.)

**Organizational department structure of Medical Technology MT and Information Technology IT within the hospital structure** We asked the hospitals for their organizational structure regarding the department of Medical Technology MT and the department of Information Technology IT. Out of all participating hospitals, the most popular answer was *separated departments* (68,8%). The major part of the UMC group also stated *separated departments* (63.6%), whereas, for the SC group, 80% of the given answers were *separated departments* in their organizational structure. 18.8% of all participating hospitals stated *subsidiary company* as their answer. Out of the group of UMCs, the answer *subsidiary company* was similar to 18.2%, and within the SC group, 20% gave the answer *subsidiary company*. The answer *joint department* was only given by two UMCs (12.5% of all inquired hospitals).

### 3.2 Category (2): degree of connectivity

**Fig. 3:**
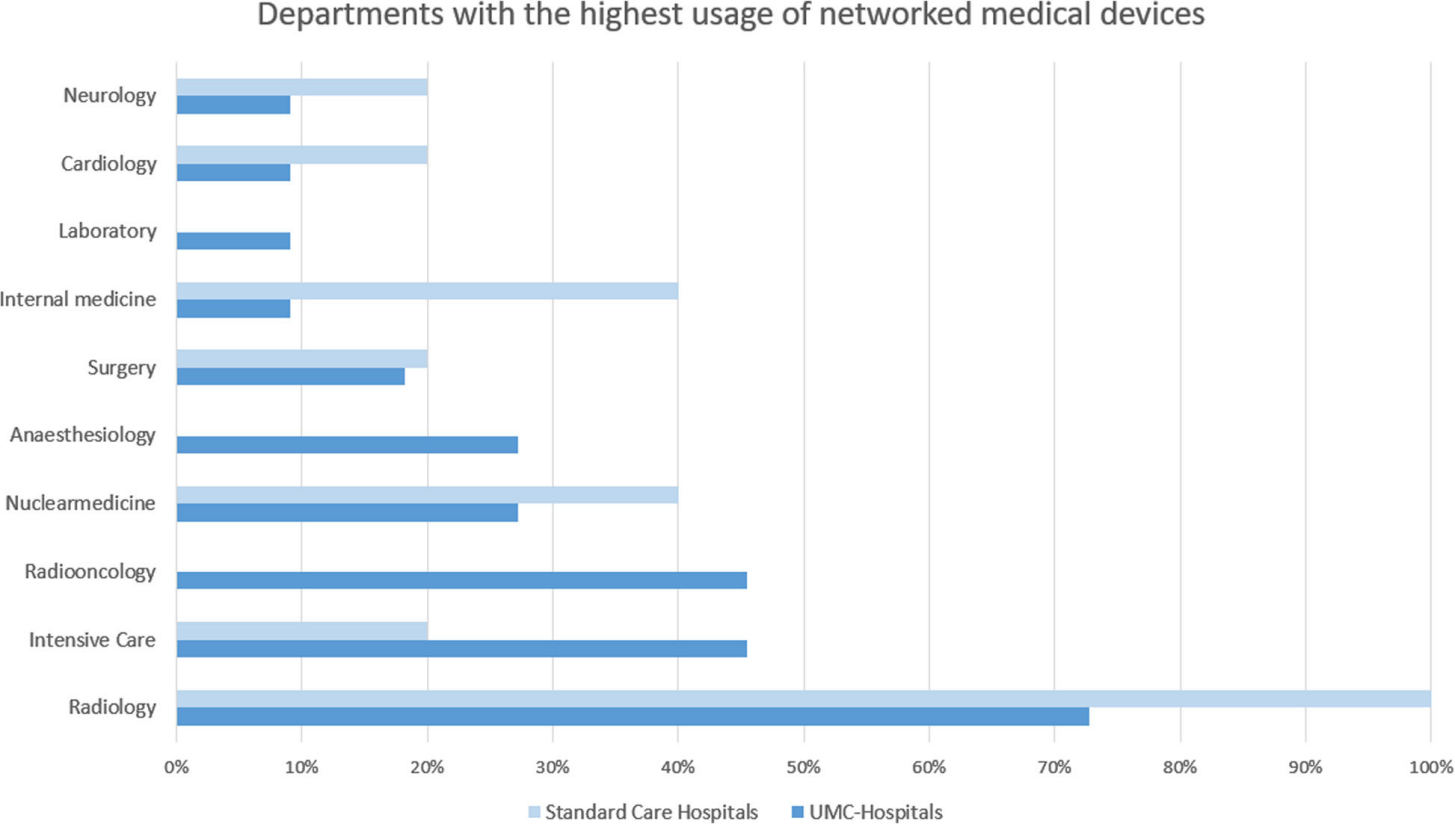
Highest usage of networked medical devices: This figure illustrates, that out of the inquired UMC hospitals, the most usage of networked medical devices can be found in departments of Radiology, Intensive care, and Radio-Oncology. The standard care hospitals are seeing their departments of Radiology, Nuclear-medicine, and Internal Medicine as the most networked

**Fig. 4:**
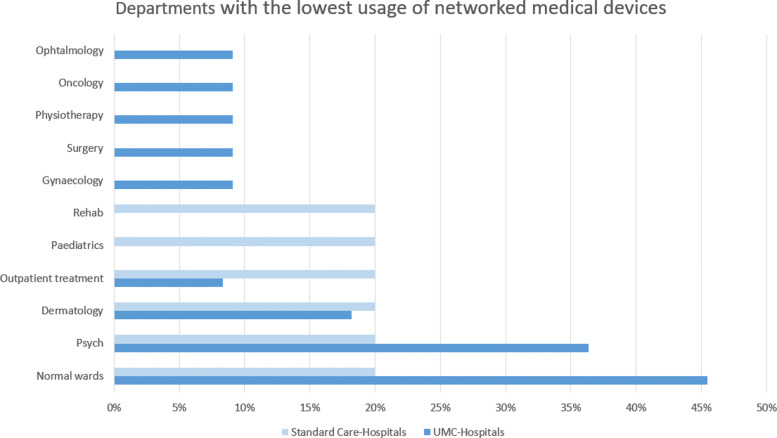
Highest growth in usage of networked medical devices in the next five years: The figure illustrates, that out of the inquired hospitals, the most expected growth can be expected in the departments of Surgery, Intensive Care, and Radiology

The results of the question regarding the department with the highest usage of networked medical devices are presented in Figure 3, and the highest estimated growth in Figure 4. The questions in this section allow multiple answers. According to the answers, the most technically specialized and therefore highly networked departments within the group of UMCs are Radiology with 73%, as well as ICU and Radio-Oncology, with 45% each. Please note that multiple answers per hospital were allowed.

Other departments with a high association with MT were mentioned as well, but less frequently: Nuclear Medicine (27%), Anaesthesiology (27%), Surgery (18%), Internal Medicine, Laboratory, Cardiology, and Neurology (all 9%). The group of SC distributed their answers more towards Radiology (100%), Nuclear Medicine (40%), and Internal Medicine (also 40%). Lower numbers were in Surgery, Cardiology, and Neurology (each with 20%).

**Fig. 5:**
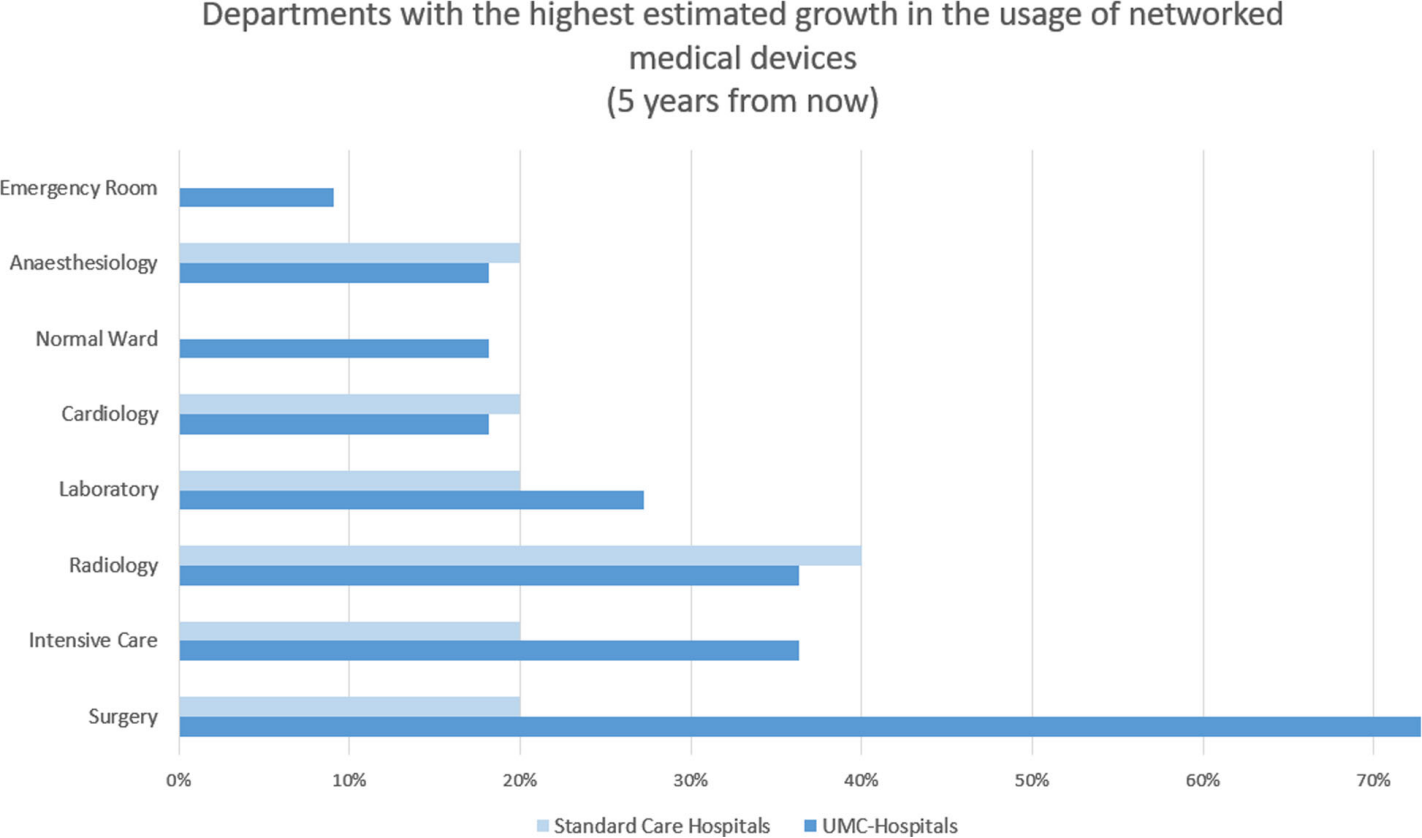
Lowest usage of networked medical devices: This figure illustrates, that out of the inquired hospitals, the lowest usage of networked medical devices can be found in normal wards in basic care as well as in disciplines of Psych, and Dermatology

**Departments with the lowest usage of networked medical devices** The results on the question of the department with the lowest usage of networked medical devices are presented in Figure 5. The most given answers in both groups were “Normal Wards in Basic Care” (UMCs 45% and SC 20%) and Psychology (UMCs 36% and SCs 20%). However, the group of SCs gave only three different types of answers, where the UMC group spread into nine different answers.

**Departments with the most expected increase in networked medical devices over the next five years**

The most expected increase in networked medical devices over the next five years is presented in Figure 4. The group of UMCs ranked Surgery with 73%, ICU and Radiology with 36% each, Laboratory with 27%, Cardiology,“Normal Wards in Basic Care” and Anaesthesiology with 18%, and Emergency Room with 9%.

### 3.3 Category (3): cybersecurity incident procedures

**Protection against cybercrime targeting networked medical devices** In these questions, the representatives were asked to rate the hospital’s protection against cybercrime targeting their networked medical devices. The group of UMCs answered with a mean of “between intermediate and good” (2.5). The group of SCs answered with an arithmetic mean of “good” (2.2). Out of the 16 hospitals, six were attacked until June 2019. Three of these attacks proofed to be successful in compromising the availability, credibility, or integrity of medical data. Six hospitals had a security incident. Out of these, three proofed to be an endangerment to the confidentiality, integrity, or availability. The networking degree of the active medical devices and the experienced attacks correlated with a coefficient of 0.53. This suggests a proportional relation between the connectivity of medical devices and the likelihood of an attack.

**Meaningfulness of insurance against the consequences of a Cyber Security Incident.** As nowadays, insurance against the consequences of an IT security incident become more apparent, 69% rated such insurance as reasonable. Table 3 shows the answers in correlation to past security incidents.

**Table 3:** This table presents the results of the interviews regarding a IT security self assessment, the attack history and the actual organisation structure within the specific hospitals

**Hospital**	**security rating (1-5 lowest)**	**Security Incident**	**Endangered CIA**	**Usefulness of Insurance**	**Organisation struc- ture of IT and MT**
1	2	*√*	*√*	*√*	Separated
2	2	×	×	*√*	Separated
3	2	×	×	*√*	Separated
4	2	×	×	*√*	Subsidiary
5	4	×	×	*√*	Separated
6	2.5	×	×	×	Separated
7	2	×	×	×	Separated
8	2.5	×	×	*√*	Combined
9	2	×	×	×	Separated
10	1	*√*	*√*	×	Subsidiary
11	5	*√*	×	*√*	Combined
12	3	*√*	×	*√*	Separated
13	2	*√*	×	×	Separated
14	2.5	×	×	*√*	Subsidiary
15	2.5	*√*	*√*	*√*	Separated
16	1	×	×	*√*	Separated

## Discussion

### 4.1 Discussion of the data set

As described in “Results” section, out of 50 hospitals, 20 representatives answered our inquiry. Initially, we presumed a higher response rate in the group of University Medical Centers UMCs. Due to their higher financial resources for IT, these hospitals possess expertise as well as skilled human resources in security associated issues. Additionally, they are bound to clinical research resulting in the assumption that the response rate to a security-related research interview would be higher than the rate of Standard Care Hospitals SCs.

Out of the 50 asked hospitals, 36 were UMCs, and 14 were SCs. Our final data set contains 11 UMCs and five SCs. This shows a response rate of 31% in the group of UMC, while the rate of SCs is 35%. We did not have any withdrawing or drop out hospitals in our survey. The achieved response rate matches the literature, where 50% or less is often a result within written surveys [26, p.95].

Interestingly, out of the 20 responsive hospitals, one hospital stated that they want to participate but did not have the required data, especially regarding the number of (networked) medical systems. This hospital is a UMC and also a member of Critical Infrastructure Protection CIP and therefore bound to the following regulations. The industry-specific standard for health care, the B3S, states in section 7.5 that networked devices must be inventoried to establish cyber security in every CIP hospital [27]. That leads to the conclusion that the hospital will not be able to assess cyber security risks as this requires knowledge of their IT landscape.

Some of the representatives did not stick with the prescribed answering options during the interview. In this case, the given answers were adapted to the standard prescribed options. This procedure contains a chance of bending the answers towards the options. We consider this deviation as insignificant because it showed in the course that the adaptation of the answers did not change the meaning.

### 4.2 General hospital features

As shown in Figure 1 and Table 2, the arithmetic average number of medical devices in the group of UMCs amounts to 25.150 devices. Furthermore, Figure 1 shows the number of networked medical devices. As the networking degree of three hospitals significantly differs from the rest, the use of the median is more reliable in this case. We reached a median value of 3.600 (14,3%) networked devices within the group of UMCs. The arithmetic average amounts to 4.500 (17,9%).

One large factor regarding the diversification of the networked numbers, is the first hospital in Figure 1. That can be described by the status of this hospital as it is one of the top three hospitals in Germany. This implies a higher capability regarding expertise and financial aspects to improve the quality of treatment. But a networking degree of 40% is considered as to high to be plausible.

The Dean-Dixon criterion shows that this value could be treated as an outlier. Excluding this hospital would result in a lower standard derivation of ± 2.860 within the networked medical device analysis.

As described above, we assume that the size of a hospital influences the network degree as the financial aspects and the available Information Technology IT expertise are higher than in small hospitals. Additionally, clinical research requires access to state-of-the-art devices that are network-capable by default nowadays [28].

Looking into the relationship of the network degree percentage and the number of medical devices reveals a strong positive correlation that can be described with a correlation coefficient of 0.53. This supports our assumption.

In Fig. 2, the number of medical devices per case is described. A majority of UMCs show a range of 0.4 to 0.6 devices per case. As these UMC hospitals are economizing similar and the quotient of devices per case is stable, this can be interpreted as an indicator for the plausibility of our data set.

Since 2013, the German regulation *SGB V paragraph 136b(1) sentence 3* obligates every UMC to publish quality reports on an annual basis. These reports contain information about their inpatient and outpatient numbers. Aggregating these reports results in an arithmetic average patient number per UMC of about 70.000 inpatients and about 317.000 outpatients.

**Organization structure of the department of Medical Technology MT and IT within the hospital structure** In the past, IT systems were only used for administrative tasks as well as documentation of patient data, diagnoses, and treatments [29]. In this time, medical devices were stand-alone and designed to particular use-cases. Therefore, MT was maintained by specialized departments. In the past 20 years, more and more medical devices interact with either each other or even with IT systems like servers and data storages [30].

This trend leads to the assumption that the department of MT has to deal with increasing IT-related matters. Furthermore, the department of IT has to integrate more MT-devices into their networks and systems. As a result, these two departments have to work together in order to guarantee proper operation. This could even include the fusion of both departments into a single one.

Contrary to our assumption, only two hospitals (12,5,%) have a combined department of medical IT. The remaining 87,5% of the hospitals stated having Medical Technology MT, and IT separated from each other. Out of these hospitals, 30% even have a subsidiary company for MT.

Having independent departments inherit the advantage of being equally in influence in the organization structure. Some of the interviewed representatives stated, that the disadvantage of being separated is a resulting gray area of responsibilities, where it is not clear, who is responsible for operating the systems and implementing essential patches. This can also be found in the literature, where it is also possible that the two departments are not cooperating and working against each other [25, p.26]. Additionally, if the MT is operated as a subsidiary company, the strategic goals of this company may differ from the goals of the main company. Furthermore, responsibilities may not be that clear in contrast to a joint department of MIT, where the cooperation is close [28]. These statements could also be verified in our study as it occurred in several interviews.

Concluding the facts mentioned above, only an organization structure with joint departments covers the requirements of operating networked medical devices in the present and future at the full grade. The capacity of being fully in charge of the device‘s mechanical functions and the networking abilities are crucial for safe and secure operation. The department’s influence within the company is strengthened due to their combined expertise as well. Making MT or IT to a subsidiary company of the main company produces specific problems when it comes to working in interdisciplinary fields like cyber security [31].

Nevertheless, as MT and IT are historically separated, it is hard to merge two existing departments inside a complex organization [25]. This can be seen as one of the reasons for the measured results.

### 4.3 Networking degree

#### 4.3.1 Highest usage and highest estimated growth potential of networked medical devices

In the following subsection, the highest usage, as well as the highest estimated growth potential of networked medical devices, is described per department or discipline. An overview of the answers to both of the discussed questions can be found in Fig. 3 and Fig. 4.

**Department of Radiology** As the department of Radiology is based on digital imaging technologies, digitization is a substantial part of it [32]. Examples of active radiological devices are: CAT-scanner-systems, MRI-systems, angiographic-systems and standard X-Ray-systems. Picture Archiving and Communication Systems (PACS) are also considered as medical devices since their inherent purpose of diagnosing patients.

The department of Radiology is, according to the results, the largest user of networked medical devices in most of the inquired hospitals (73% in UMCs and 100% SCs).Regarding the growth in networked medical device usage in the next five years, the department of Radiology comes in third place with 36% in UMCs and 40% in SCs.

Reviewing these results leads to the conclusion that the digitization and networking process in the group of UMC is changing. The digitization and networking process in the department of Radiology seems to have progressed so far that UMCs are able to focus on extending the networking degree in other departments and disciplines.

It can also be seen in the average number of medical imaging devices per hospital (Table 4). The 34 UMCs, which are 2.3% of all hospitals in Germany operate 13.6% CAT scanners and 18.7% MRI scanners [5, p. 58].

**Table 4:** This table presents the data given from the German Federal Office of Statistics regarding the so called large-scale equipment in German hospitals and especially in UMCs. The UMCs proportion is given in percentage of the total number of devices

**Art of device**	**Total number of devices**	**Number of devices in UMC**	**percentage**
CAT-Scanner	1551	211	13.6%
MRI-Scanner	1011	189	18.7%
DSA-System	884	153	17.3%
LINAC	400	132	33%
PET-Scanner	142	44	31%

**Discipline of Intensive Care (IC)** The discipline of Intensive Care relies on the exact application of life support devices and device based patient monitoring. Examples are: Respirators, infusion pumps, monitoring systems. The capability of intensive care technology has increased in the past years [33]. This enhances a trend towards more interconnectivity and digitization in intensive care [34]. Again, we propose a pioneering role in the group of UMC in the usage of networked devices in the field of Intensive care.

Inside the group of UMCs, Intensive Care is already the second-largest user of networked medical devices, with 45% of the given answers. The SCs are not that far progressed in this matter, as 20% of SCs mentioned this department. This may also derive from the fact that the 34 UMCs cover about 18% of all intensive care capabilities in Germany, enabling these hospitals to spend more resources for intensive care technology [5, p. 68].

The inquiry of the highest growth in the usage of networked medical devices shows the departments of Intensive Care with 36% in second place in the group of UMCs.

**Department of Radio-Oncology RO**

The discipline of RO consists on the application of highly technical radiation devices. Ionizing radiation is produced either by radioactive decay or by electron acceleration within a large magnetic field. These devices are processing and generating image data to ensure a highly conformal conservation of the healthy tissue.

RO is only mentioned in the group of UMC. Due to its need for large-scale equipment, departments of RO are only found in large hospitals like UMCs resulting in 166 German hospitals operating a department of RO. Out of these 166, 32 UMCs are operating about 33% of all Radio-Oncologic linear accelerator LINACs, which are vital for the therapy [5, p.58]. This can also be seen in the results, as 45% mentioned this department sharing second place with intensive care.

**Discipline of Nuclear Medicine NUC** The discipline of NUC is another highly technology depending field of medicine. The patient’s metabolism is made visible through an uptake measurement of specific injected, gamma-ray-emitting radioactive isotopes so called tracers. These isotopes are either produced in the hospital or brought from a near nuclear reactor facility. In combination with conventional radiological procedures the NUC is essential for the correct diagnosis of oncologic patients. It depends on the cooperation of image data, the availability of radioactive isotopes, and the timed functionality of several networked devices [35].

UMC Hospitals cover a large part of the national nuclear medical care. For example, nearly one third (31%) of the total usage of Positron Emission Tomography PET systems is performed by 31 UMCs [5, p. 58]. Therefore, we assumed a high network degree in the group of UMCs.

Nevertheless, only 27% of the inquired UMCs stated this department as one of the highest users of networked medical devices. We did not expect this result. A possible reason for this could be a missing mentioning by the hospital representatives due to the inclusion of this department into the department of radiology.

To validate these results,we tried to get back to the hospital representatives with this and other outliers but unfortunately, we did not receive any answers. This particular question was discussed with the head of MT at the local UMC. He stated also that a possible explanation for this result is the inclusion of this department into the department of radiology.

**Discipline of Anaesthesiology** The discipline of Anaesthesiology relies on narcotic and life maintaining devices as well as devices to monitor the patients’ vital parameters. Historically, these devices operate on their own without any network connection. Nevertheless, the trend towards an intelligent networked operating system influences the Anaesthesiology massively [36, p.39]. The UMC group stated this discipline also with 27%. The group of SCs did not mention this discipline, but 18% of the UMCs and 20% of the SCs stated this discipline as one with high growth in the usage of networked medical devices.

**Discipline of Surgery** The discipline of Surgery was stated by 18% of the UMCs and 20% of the SCs. As mentioned above, we suspect a partial separation from the discipline of Anaesthesiology.

In matters of future growth, the discipline of Surgery leads the survey results with 73% in the group of UMC. This proofs our hypothesis that a connected smart surgery with imaging processing, navigation, robotics, and connected Anaesthesiology is one of the most anticipated steps in the UMC hospital development in the next five years. This concludes with the literature [37]. Hoeckelmann et al. presume a gradual increasing usage of robotics in a connected Surgery [38]. Additionally, 3D-printing is progressing in state of the art departments of surgery [39].

Additionally, as part of the surgery, growth in network usage regarding the emergency rooms is expected by 9% of the UMCs.

**Department of Internal Medicine** The department of internal medicine was mentioned within the SCs by 40% and the UMC group by 9%. UMCs usually cover a vast range of specialized disciplines, leading to a lot of individual departments for single disciplines, for example, cardiology or oncology. In SCs, those particular disciplines are often combined into a superior department of internal medicine [40].

Unfortunately, there were no mentions of this department in context to usage growth.

**Department of Cardiology** In Cardiology, active medical devices are used to ensure the electro-mechanic function of the patients heart. There are devices as: pacemakers and defibrillators, but also diagnostic devices as, ultrasonic-systems and radiological systems. In Germany, there are 323 specialized departments for Cardiology [5, p. 26]. Assuming that every UMC possesses a department of Cardiology, 20% of SCs operate a special department for cardiology. This leads to the conclusion that a department of Cardiology inside an SC is often part of their specialization. Looking at the result of the survey, 20% of SCs mentioned this department as one of the departments with the highest networking degree. Out of the UMCs, only 9% stated this department as it is part of their primary care. Regarding the growth in the next five years, the results of UMCs and SCs are close to each other, with 18% and 20%. This can be explained by the fact that connected devices are relatively new in the part of cardiology [41], leading to increased growth for departments in UMCs as well as in hospitals with an emphasis on cardiology.

**Department of Neurology** The department of Neurology uses highly sensible sensor technology to measure little neurologic voltage potentials [42]. To improve the workflow, currently, big data and AI concepts are on the edge of getting implemented into the clinical workflow of Neurology [43, p.8]. Additionally, Teleneurology is trending in this field of medicine [44]. Implementing these technologies requires high connectivity between the used medical devices.

This department was mentioned by 9% of the UMCs as one of the most significant users of networked medical devices but did not appear in the departments of highest growth. That refutes the thesis but shows that some UMCs are focusing on Neurology as one of their core competences. One of the SCs (20%) stated this department leading to the assumption that this hospital is specialized in Neurology.

**Discipline of Laboratory** The discipline of Laboratory is an essential part of the clinical workflow. Fast and correct treatment of patients mainly depends on the exactness as well as the timely availability of the results. This can be achieved by using connected systems processing data automatically and making it available to all stakeholders directly [45]. Additionally, a trend in Laboratory is the so-called “In-Home” laboratories, where patients have devices at home, sending data to the treating physician [46]. Laboratory equipment is therefore also diagnostic equipment, the size of which depends on the number of cases treated.

The discipline of Laboratory is mentioned as one of the most significant users of networked medical devices (9%) in the group of UMCs.

Further, it is considered with 27% (UMC) and 20% (SC) as one discipline with the highest future potential in becoming a large user of networked medical devices. This matches our expectations of laboratory devices, achieving a faster work process operating automatically and connected with other systems.

**Normal Wards in Basic Care** Normal wards in basic care cover every inpatient not being in intensive or intermediate care. It is part of most of the treating disciplines inside a hospital and therefore considered as one of the most abundant components in basic treatment.

Being one of the largest parts in a hospitals’ basic proficiency, it is also one of the least connected disciplines, as can be seen in Fig. 5.

Nevertheless, the possibilities of using network-connected devices to improve and support normal wards in basic care are increasing. There are two main use cases for connected devices on normal wards to improve individual treatment: Connected patient beds [47] and the generation of real-time data from patients [48]. The connected patient bed can, for example, be used to display and manage the patient’s digital medical record. Additionally, the connected bed could be extended by measuring units supporting the second part, the real-time data generation.

This proposition implies an expected growth in the networking connectivity of normal ward’s devices, which is consistent with the results having 18% of the UMCs mentioning this department.

#### 4.3.2 Lowest usage of networked medical devices

In the following subsection, the lowest usage of networked medical devices is described per department or discipline. An overview of the answers to the discussed questions can be found in Fig. 5.

Comparing the results with the preceding ones, a more extensive range of answer distribution was recognized.

**Normal Wards in Basic Care** As mentioned in the last section, the normal wards in basic care are mentioned as the lowest connected discipline. This was mentioned by 45% of all UMCs.

However, global digitization and networking processes like the implementation of an electronic patient record are progressing into this discipline [49].

**Discipline of Psychology** The discipline of psychology is not widely based on the usage of medical devices [50]. Therefore, the total number of networked medical devices in this area is limited. This can also be seen in the answers of the hospitals as 36% of the UMCs, and 20% of the SCs mention this as one of their lowest networked discipline.

**Discipline of Dermatology** This discipline relies heavily on the usage of microscopic and imaging devices, as well as therapy illuminants, which fall under the classification in this subject area as active medical devices. [51]. These devices are still running as stand-alone devices without a networked component. The group of UMCs stated this department with 18% as one of their lowest networked departments. The SCs stated 20% for this department.

Connecting those devices could support the workflow inside the dermatology and is requested by a majority of patients who could benefit from such development as, e.g., the inclusion of apps to screen lesions in matters of malicious melanoma [52].

**Departments with the lowest usage of networked medical devices** The answers to this particular question resulted in a high range. The most common answer was “Normal Wards in Basic Care”, with 40%. This leads to the assumption that in normal wards in basic care, networked medical devices are not more attractive to use, than the analog variant. A possible cause for this can also be seen in the fact, that the implementation and usage of digital devices is considered frustrating if the underlying data and infrastructure in the organisation is not compliant[23]. We can associate this measure with a high degree of representativeness, since all of the hospitals surveyed have normal wards in basic care.

Due to individual clinical politic in setting the focus on different departments, we received a wide variety of answers. Many of these departments are historically present in the clinic and mainly operate without the use of networked MT. 25% of the inquired hospitals stated the department of dermatology as the one with the lowest usage of networked medical devices. This connects to the assumption that even in the most sophisticated hospitals in Germany, the major part of the used devices are still without the usage of any network. The nomination of the department of ophthalmology suggests a major usage of analog optical systems in both departments. The absence of a universal digital image standard like DICOM for radiological images is evidently.

### 4.4 Emphasis on networked medical devices in the next five years

The literature shows that the increase in the amount of networked medical devices is one of the essential improvements in medical care during the next years.[37, 53–55]

Two of the inquired hospitals did not want to give an answer to this question in the shortcoming of an official opinion. Ten out of eleven UMCs answered this question with *high*. This is in line with our expectations. Some of the representatives even increased the voting of *high* to *enormous* and *gigantic* in specific departments Fig. 4. We can state that a major part of German UMCs is about to increase their percentage of networked medical devices significantly in the next five years.

### 4.5 Information security status in hospitals

**Protection against cybercrime targeting networked medical devices** The majority of the inquired hospital representatives stated their hospital as *intermediate-well protected* against cybercriminals targeting their networked medical devices.

However, the representatives are under some pressure not to portray the hospital as unfavorable due to public relations causes. This glossing over the own company’s security assessment over the average sector value is known in other Critical Infrastructure Protection CIP branches as well [21, p.19].

Some of the representatives were concerned about their missing knowledge and limited resources to operate the systems on a more secure basis. This, in combination with the fact above, leads to the assumption that the true self-assessment of the hospitals is more likely to be less secure than promoted in our interview. This can also be seen in related literature, where 50.5% of all inquired CIP institutions suffered from an cyber security incident in 2017. The overall security assessment against this kind of risk was stated as *high* within 58% of the inquired institutions [21].

Our results show a total attack rate of 37.5%. The group of UMCs was attacked with a rate of 27.2% and the SCs with 80%.

We propose a relationship between the large pool of networked medical devices and the attack surface of UMCs [11, pp.637]. This is supported by our measurement of attacks and the correlating degree of connectivity (0.53). This correlation is considered as strong positive. It is based on our complete hospital data not only looking at the UMC hospitals. Further, we anticipate a specific influence of the existing organization structure in operating these devices in a secure way. Therefore, we correlated the cyber security self-assessment and the successfully performed attacks with each other and reached a coefficient of -0.27. This is considered to be a small to medium correlation. We also looked into the stated organization structure and the successfully performed attacks on networked medical devices, and it became obvious that all successfully performed attacks happened in hospitals with either separated or subsidiary departments of MT and IT. Reviewing the successful and unsuccessful performed attacks, we discovered a success rate of 67% within the group of attacked UMCs and a rate of 33% within the attacked SCs. As the data set is limited, this result is a qualitative statement.

**Meaningfulness of insurance against the consequences of an IT security incident** 69% of the inquired hospitals would consider an insurance against the consequences of an cyber security incident in their hospital. This implies a big concern in matters of cyber security. Comparing the assessment with regard to the vulnerability and the meaningfulness of an insurance, one does not recognize any correlation. Even if the hospital representatives consider their hospital as well protected against cybercrime, they would still recommend placing an insurance against these kinds of threats as residual risk coverage. Furthermore, this supports the thesis that there could never be an absolute security level [56].

## Conclusions

The data collected from the German hospitals of maximum care shows a diverse situation in some aspects of IT security.

German University Medical Centers UMCs are facing a substantial change within their landscape of medical devices becoming more and more networked. The conducted survey showed a median of 25.500 medical devices and containing a median of 3600 networked medical devices. That results in a strong correlation between the actual number of medical devices and the networking degree of 0.53.

The departments of Radiology, Intensive Care, Radio-Oncology, and Nuclear Medicine are currently the largest users of networked medical devices. In the next five years, the usage of networked medical devices will increase significantly in the departments of Surgery, Intensive Care, and Radiology. That could lead to a growing attack surface in matters of cyber security.

Concluding answers regarding the cyber security status reveals a lack of security basics in some of the inquired hospitals. Some representatives even were concerned about their missing knowledge and limited resources to operate the systems on a more secure basis. However, that does not align with the given Security self-assessment.

Most of the inquired hospitals are operating separated departments of IT and MT. The literature and our experience show problems in handling cyber security incidents on the organizational level. As a result, the most suitable organization structure for handling the requirements of operating networked medical devices is a joint department with a combination of both fields of expertise. We did also discover, that success-full attacks only happened in hospitals with separated departments of MT and IT.

Cyber security in German healthcare might become a major issue. We detected a strong positive correlation (0.53) between the state of being attacked and the networking degree of active medical devices. Consequently, some of the inquired hospitals are already facing the consequences of omitted measures within their growing pool of medical devices. Continuously relying on historically grown structures without adaption and trusting manufactures to solve vectors is a critical behavior that could seriously endanger patients. Some hospitals are already on a good path here, and talking to the responsible persons showed much awareness for this topic, but a common, nationwide approach is still missing.

## Supplementary information

**Additional file 1** The specific questionnaire used for the interviews.

## Data Availability

The data sets generated and analysed during the current study is available from the corresponding authors on reasonable request.

## Abbreviations

CIP: Critical Infrastructure Protection
IT: Information Technology
LINAC: Radio-Oncologic linearaccelerator
MT: Medical Technology
NUC: Nuclear Medicine
PET: Positron Emission Tomography
RO: Radio-Oncology
SC: Standard Care Hospital
UMC: University Medical Center
